# Case Report: Benign Infantile Seizures Temporally Associated With COVID-19

**DOI:** 10.3389/fped.2020.00507

**Published:** 2020-08-06

**Authors:** Marcos García-Howard, Mercedes Herranz-Aguirre, Laura Moreno-Galarraga, María Urretavizcaya-Martínez, Josune Alegría-Echauri, Nerea Gorría-Redondo, Laura Planas-Serra, Agatha Schlüter, Marta Gut, Aurora Pujol, Sergio Aguilera-Albesa

**Affiliations:** ^1^Department of Pediatrics, CHN, Navarra Health Service Hospital, Pamplona, Spain; ^2^Pediatric Infectious Disease Unit, Department of Pediatrics, CHN, Navarra Health Service Hospital, Pamplona, Spain; ^3^NavarraBioMed and IdiSNA, Health Research Institute, Pamplona, Spain; ^4^Pediatric Respiratory Medicine, Department of Pediatrics, CHN, Navarra Health Service Hospital, Pamplona, Spain; ^5^Pediatric Neurology Unit, Department of Pediatrics, CHN, Navarra Health Service Hospital, Pamplona, Spain; ^6^Neurometabolic Diseases Laboratory, Bellvitge Biomedical Research Institute (IDIBELL), Barcelona, Spain; ^7^Center for Biomedical Research on Rare Diseases (CIBERER), ISCIII, Madrid, Spain; ^8^CNAG-CRG, Centre for Genomic Regulation, Barcelona Institute of Science and Technology, Barcelona, Spain; ^9^Universitat Pompeu Fabra (UPF), Barcelona, Spain; ^10^Catalan Institution of Research and Advanced Studies (ICREA), Barcelona, Spain

**Keywords:** coronavirus, SARS-CoV-2, COVID-19, pediatric COVID-19, non-febrile seizures, afebrile seizures, PRRT2 mutations, benign familial infantile epilepsy

## Abstract

**Background:** Non-febrile illness seizures may present in previously healthy children as afebrile seizures associated with minor infections, such as mild gastroenteritis or respiratory tract infections, and are linked to a genetic predisposition. For the novel human coronavirus SARS-CoV-2, causing COVID-19, fever, cough, and gastrointestinal complaints are the most common symptoms in children, and a hyperimmune response may be present. No detailed temporally associated neurological complications have been documented in pediatric case series so far.

**Case description:** We present the case of a 3-months-old girl with non-febrile repeated seizures in a COVID-19 family setting. The infant started with a mild fever and cough that lasted for 2 days. At day 6 from onset, the girl presented with two focal motor seizures with impaired consciousness and awareness. All investigations ruled out signs of meningo-encephalitis or active epilepsy, including normal electroencephalogram and cerebral magnetic resonance imaging. PCR from nasal and throat swabs was positive for SARS-CoV-2. Remarkably, blood ferritin and D-dimer levels were increased. At day 9, the infant presented another afebrile motor seizure, and levetiracetam dose was modified there was a favorable response within 3 months of the follow-up. Much interest has been raised with regards to host genetic determinants to disease severity and susceptibility to COVID-19. We thus performed whole exome sequencing, revealing a pathogenic frameshift mutation in the PRRT2 gene in both the mother and the infant. The mother had presented two late infantile febrile convulsions with normal outcome afterwards.

**Discussion:** The hyperimmune response described in adult cases with COVID-19 can be seen in infants, even in the absence of respiratory symptoms. Moreover, COVID-19 may present in infants as non-febrile seizures, triggering early onset seizures in infants with a genetic predisposition. In this pandemic situation, precision medicine using massive sequencing can shed light on underlying molecular mechanisms driving the host response to COVID-19.

## Introduction

Non-febrile illness seizures are described as afebrile seizures associated with minor infections in previously healthy children. Seizures occur mainly in infants in the setting of acute infections, such as mild gastroenteritis or respiratory tract infections, without structural correlate or hydro-electrolytic imbalance (1–4). Rotaviruses are frequently found in non-febrile convulsions associated with gastroenteritis, and noroviruses have been recently identified as an emergent pathogen in these cases (1, 5). In infants with non-febrile seizures related to respiratory tract infections, common seasonal viruses, such as influenza, respiratory syncytial virus (RSV), and metapneumovirus, have been pointed out as plausible causative pathogens (3, 4, 6).

Human coronavirus (HCoV) causes respiratory infections with a seasonal pattern in children, and in some cases, extra-pulmonar manifestations have been described. It has been increasingly recognized that HCoV shows some neurotropism due to its capacity to reach the central nervous system after the nasal infection, shown for HCoV-OC43 and HCoV-NL63 (7, 8). Neurological complications of common HCoV infections have been reported, including febrile seizures, convulsions, loss of consciousness, encephalomyelitis, and encephalitis (9). Another HCoV, SARS-CoV, emerged in Guandong province, southern China, in 2002, and it spread to many countries and caused severe lower respiratory tract infection with an overall case-fatality rate of 10%. The SARS-CoV was associated with milder disease in children compared to adults, with some case series reporting febrile seizures in 10% of a total sample of 41 children (10–12). Fortunately, no human SARS-CoV infections have been identified since July 2003 (8).

In December 2019, a novel HCoV (SARS-CoV-2) was reported from Wuhan city, Hubei province, China, and it rapidly spread worldwide causing a pandemic outbreak by March 2020, producing a respiratory disease called COVID-19. Initial case series have shown that children present milder clinical symptoms than adults and that most pediatric cases were infected in family clusters. Fever, cough, respiratory distress, myalgia, and gastrointestinal complaints are the most common symptoms (13–18), but no detailed neurological complications have been documented in pediatric case series so far.

In this article, we describe the case of a 3-months-old girl with non-febrile repeated seizures in a COVID-19 family setting. Whole exome sequencing was applied and revealed an underlying genetic pathogenic variant that may cause the clinical presentation.

## Case Presentation

A previously healthy, with uneventful pregnancy and delivery, 3-months-old girl was admitted to the pediatric emergency department early morning on April 1 after her mother reported two episodes of convulsions without fever. During the night, the mother, who was a nurse, reported a first episode of clonic movements of the face with tonic posture of extremities and trismus, without consciousness, lasting 3 min approximately. Few hours later, the infant presented with a second episode, described as staring gaze, clonic movements of the face and right extremities, and repeating sucking movements of the mouth, lasting <5 min. At admission, vital constants were normal; physical and neurological examination showed mild hypotonia and drowsiness without focal deficits. The mother informed us that, on March 27–28, the infant had presented with a low fever of <38.1°C, rhinorrhea, cough, and diarrhea with subsequent improvement. No fever was documented the 3 days before these convulsions. Interestingly, the mother referred herself as having persistent symptoms of anosmia and dysgeusia since March 23, and showed no signs of fever or respiratory symptoms since.

At admission, patient blood tests did not show any abnormalities except for a high ferritin value (385 μg/L; normal values 10–204). PCRs of nasopharyngeal and throat swabs tested positive for SARS-CoV-2. Additional testing for other viruses was negative, including HCoV-NL63, HCoV-OC43, HCoV-229E, RSV, rhinovirus, metapneumovirus, influenza, adenovirus, bocavirus, and enterovirus. Chest x-ray and brain CT scans did not reveal abnormalities; the CSF analysis for cells, glucose, and protein was normal. PCRs for herpes virus family (HSV-1, HSV-2, and VZV) and enterovirus in CSF were negative. Bacteria cultures in blood, urine, and CSF were also negative.

**Figure 1:**
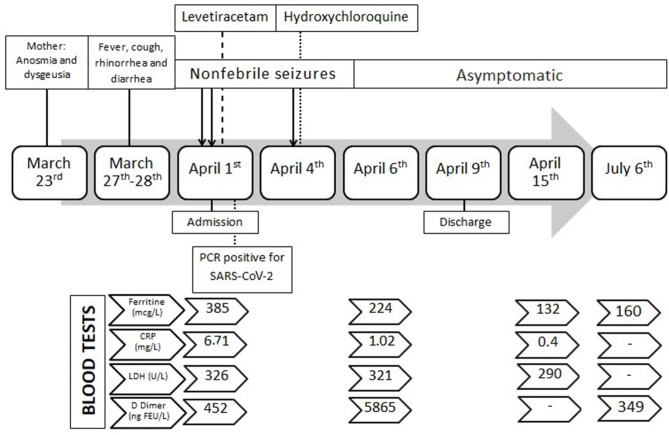
Timeline course of the patient's COVID-19 disease.

During hospitalization, three interictal electroencephalograms (EEG) and a cerebral 1.5T MRI showed normal results. Levetiracetam was started the 1st day as prophylactic antiepileptic treatment (28 mg/Kg/day), but the infant presented with another afebrile seizure on April 4, consisting of upright tonic eye deviation, clonic movements of face muscles, and tonic posture of four limbs in extension, lasting 90 s. The seizure was recorded by the mother and was checked by pediatric neurologists. Pre-dosing blood levels of levetiracetam were within therapeutic range (15 mcg/mL; normal values 10–40). Hydroxychloroquine was then started on April 4, as compassionate use due to the persistence of seizures in a COVID-19 setting, at a dosage of 6.5 mg/Kg/day for 5 days with excellent tolerance. Blood test controls revealed a sustained decrease of ferritin values, with a late increase of D-dimer levels. Unfortunately, D-dimer control was not possible due to technical procedures. The rest of the lab tests were within normal limits. Following discharge, the patient was followed as an outpatient, and she presented with normal neurological development and an absence of seizures. A timeline of clinical course, lab test, and investigations is summarized in Figure 1. Informed and written consent to publish clinical details was obtained from the parents. Additionally, during hospitalization, the patient and her mother were included in a collaborative study of genomic medicine for identifying genetic variants causing hyperimmunity due to SARS-CoV-2 infection. The local ethics committee approved the study. Whole exome sequencing was performed, and prioritized genes were analyzed. No pathogenic variants of susceptibility genes for hyperimmunity were found, but both the infant and the mother carried a loss-of-function variant in the PRRT2 gene (NM_145239.3), c.649dupC (p.Arg217fs), at the heterozygous state. This frameshift variant has been recurrently described in ClinVar as pathogenic. It is associated with benign familial infantile convulsions (OMIM 605751), but it is also allelic to infantile convulsions and choreoathetosis (OMIM 602066) (19). A revision of maternal family history revealed that the mother could have had two convulsions during late infancy, related to mild infections and fever, with normal development afterwards.

## Discussion

SARS-CoV-2 infection in children is being increasingly recognized. However, detailed clinical data are still lacking and individual cases, such as the one presented, can shed some light to comprehend the complex systemic manifestations of this disease in the youngest.

A review of the Chinese Center for Disease Control and Prevention on February 24 has shown than <1% of the COVID-19 cases were in children younger than 10 years of age (20). In Wuhan Children's Hospital, China, 1,391 children were tested through February, and a total of 171 (12.3%) were positive for SARS-CoV-2 infection, with a median age of 6.7 years. Of these, 65% presented pneumonia and three required invasive mechanical ventilation. Only 18% of the positive children were infants younger than 1 year of age, and no cases were reported with neurological features. In Spain, by March 16, 41 of the 4,695 confirmed cases (0.8%) in the Madrid region were children younger than 18 years, and 60% of the pediatric cases required hospitalization (21). In a systematic review of SARS-CoV-2 infection in children, Castagnoli et al. found, by April 22, 444 participants younger than 10 years of age, but no details about clinical symptoms were revealed (22).

Our infant presented with afebrile seizures during the course of COVID-19, some of them with focal semiology, and these were not associated with signs of encephalitis, structural damage, or other concomitant infection. The clinical picture is compatible with the definition of non-febrile illness seizures, which occur in association with an acute infection the week before or 3 days after the seizure, although without presenting with fever on the day of the seizure (3, 23).

Besides respiratory symptoms, HCoV infections may present with febrile seizures in susceptible infants (9–12). Very recently, a febrile convulsion in a 2-years-old girl with COVID-19 was reported (15). However, several studies have suggested that non-febrile illness seizures are a different seizure category from febrile seizures or unprovoked seizures. Non-febrile illness seizures may share some genetic predisposition in a similar manner as febrile seizures or epilepsy, and, as with febrile seizures, the prognosis is favorable in most cases (3).

This pandemic has sparked an interest in genomic medicine to elucidate host determinants of phenotype severity (24, 25). We thus performed whole exome sequencing as described (26) and uncovered the genetic predisposition of the infant to develop afebrile seizures due to a well-known recurrent pathogenic PRRT2 mutation associated with benign familial infantile convulsions (OMIM 605751) and infantile convulsions and choreoathetosis (OMIM 602066) (19). In the mother, the phenotype is benign and self-limited, without movement disorders. Nonetheless, a long-term follow-up is required to detect the possible development of dyskinesias in the infant.

Regarding other investigations in this case, ferritin levels were increased at admission, on day 6 since symptoms onset, and they progressively decreased over time. D-dimers were within normal limits at admission but increased during hospitalization. These findings resemble the hyperimmune response found is adult COVID-19 severe cases, a major driver of adverse outcome (27, 28). Same findings are being reported in severe COVID-19 in children (8), and, during the SARS-CoV epidemic of 2002, some patients presented with decreased lymphocyte count and increased levels of LDH and D-dimers.

Therapeutic strategies and evidence-based protocols for COVID-19 treatment in children are still lacking. On April 4, we thus decided to treat our infant with hydroxychloroquine since this drug is renowned for its antiviral and immunomodulating properties (29), and it has been previously used in young infants (30–33). Preliminary results of hydroxychloroquine on adults COVID-19 clinical trials suggested that 600 mg daily may decrease viral load in nasal swabs (34), but further studies has raised concerns about its use during hospitalization (35). On July 4, the WHO International Steering Committee discontinued clinical trials for hydroxychloroquine in hospitalized patients (www.who.int/news-room).

One limitation of this case is related to the storing of the biological samples. PCR of SARS-CoV-2 in CSF was not available at that moment, and the sample was not stored. Moreover, hyperimmune response in younger children is an uncommon phenomenon and warrants further research in samples of the patients. We would like to encourage pediatricians to collect and store biological samples of patients with COVID-19 for further analysis, as it would be useful for future research.

## Data Availability Statement

The original contributions presented in the study are included in the article/supplementary material, further inquiries can be directed to the corresponding author/s.

## Ethics Statement

The studies involving human participants were reviewed and approved by Complejo Hospitalario de Navarra. Written informed consent to participate in this study was provided by the participants' legal guardian/next of kin. Written informed consent was obtained from the minor(s)' legal guardian/next of kin for the publication of any potentially identifiable images or data included in this article.

## Author Contributions

SA-A and MH-A conceived and designed the manuscript. MG-H wrote the first draft. LM-G, MU-M, JA-E, and NG-R provided data acquisition, clinical details of the patient, and reviewed the literature. LP-S, AS, and MG generated and analyzed the exomes using the CNAG pipeline. AP provided funding and interpreted the variants in its clinical context. SA-A wrote the final manuscript. AP and SA-A added and refined discussion and final conclusions. All authors reviewed and approved the final version as submitted, and agreed to be accountable for all aspects of the work.

## Conflict of Interest

The authors declare that the research was conducted in the absence of any commercial or financial relationships that could be construed as a potential conflict of interest.
